# Week 96 Genotypic and Phenotypic Results of the Fostemsavir Phase 3 BRIGHTE Study in Heavily Treatment-Experienced Adults Living with Multidrug-Resistant HIV-1

**DOI:** 10.1128/aac.01751-21

**Published:** 2022-05-03

**Authors:** Margaret Gartland, Pedro Cahn, Edwin DeJesus, Ricardo Sobhie Diaz, Robert Grossberg, Michael Kozal, Princy Kumar, Jean-Michel Molina, Fernando Mendo Urbina, Marcia Wang, Fangfang Du, Shiven Chabria, Andrew Clark, Louise Garside, Mark Krystal, Frank Mannino, Amy Pierce, Peter Ackerman, Max Lataillade

**Affiliations:** a ViiV Healthcare, Research Triangle Park, North Carolina, USA; b Fundación Huesped, Buenos Aires, Argentina; c Orlando Immunology Center, Orlando, Florida, USA; d Infectious Diseases Division, Paulista School of Medicine, Federal University of São Paulo, São Paulo, Brazil; e Montefiore Medical Center, Bronx, New York, USA; f Department of Internal Medicine, Infectious Diseases Section, Yale University School of Medicine, New Haven, Connecticut, USA; g Department of Medicine and Microbiology, Georgetown University Medical Center, Washington, DC, USA; h University of Paris, Saint-Louis and Lariboisière Hospitals, Assistance Publique Hôpitaux de Paris, Paris, France; i Hospital Nacional Edgardo Rebagliati Martins, Lima, Peru; j GlaxoSmithKline, Collegeville, Pennsylvania, USA; k ViiV Healthcare, Branford, Connecticut, USA; l ViiV Healthcare, Brentford, United Kingdom; m GlaxoSmithKline, Brentford, United Kingdom

**Keywords:** fostemsavir, heavily treatment experienced, multiple antiretroviral drug resistance, attachment inhibitor, HIV-1, optimized background therapy, antiretroviral agents

## Abstract

In the phase 3 BRIGHTE study in heavily treatment-experienced adults with multidrug-resistant HIV-1, fostemsavir plus optimized background therapy (OBT) resulted in sustained rates of virologic suppression through 96 weeks. HIV-1 RNA <40 copies/mL was achieved in 163/272 (60%) Randomized Cohort (RC) participants (with 1 or 2 remaining approved fully active antiretrovirals) and 37/99 (37%) Non-randomized Cohort (NRC) participants (with 0 fully active antiretrovirals). Here we report genotypic and phenotypic analyses of HIV-1 samples from 63/272 (23%) RC participants and 49/99 (49%) NRC participants who met protocol-defined virologic failure (PDVF) criteria through Week 96. The incidence of PDVF was as expected in this difficult-to-treat patient population and, among RC participants, was comparable regardless of the presence of predefined gp120 amino acid substitutions that potentially influence phenotypic susceptibility to temsavir (S375H/I/M/N/T, M426L, M434I, M475I) or baseline temsavir 50% inhibitory concentration fold change (IC_50_ FC). The incidence of PDVF was lower among participants with higher overall susceptibility score to newly used antiretrovirals (OSS-new), indicating that OSS-new may be a preferred predictor of virologic outcome in heavily treatment-experienced individuals. Predefined gp120 substitutions, most commonly M426L or S375N, were emergent on treatment in 24/50 (48%) RC and 33/44 (75%) NRC participants with PDVF, with related increases in temsavir IC_50_ FC. In BRIGHTE, PDVF was not consistently associated with treatment-emergent genotypic or phenotypic changes in susceptibility to temsavir or to antiretrovirals in the initial OBT. Further research will be needed to identify which factors are most likely to contribute to virologic failure in this heavily treatment-experienced population (ClinicalTrials.gov, NCT02362503).

## INTRODUCTION

Despite the success of combination antiretroviral therapy for the treatment of HIV-1, virologic failure remains a problem for some individuals, increasing the risk of drug resistance; limiting future treatment options; and impacting morbidity, mortality, and health care burden (1–3). For some heavily treatment-experienced (HTE) people living with multidrug-resistant HIV and/or limitations resulting from toxicities and/or intolerance to antiretroviral drugs, forming a suppressive combination antiretroviral regimen is not feasible (3–5). For these individuals, there is a continued need for new classes of antiretroviral drugs with novel mechanisms of action that are well tolerated and lack cross-resistance to currently available therapies (3–6).

Fostemsavir (Rukobia; ViiV Healthcare, Research Triangle Park, NC), a prodrug of the first-in-class attachment inhibitor temsavir, is indicated in combination with other antiretrovirals for the treatment of multidrug-resistant HIV-1 infection in HTE adults with limited antiretroviral treatment options (7–11). Temsavir has a novel mechanism of action, binding directly to the viral envelope gp120 at a conserved site under the β20-21 loop, close to the CD4 binding site, locking gp120 into a closed state that prohibits the conformational change necessary for initial interaction between the virus and cell-surface CD4 receptors, thereby preventing binding to and entry into host CD4^+^ T cells and other immune cells (7, 12). Temsavir has demonstrated no *in vitro* cross-resistance with other antiretroviral classes, including ibalizumab, maraviroc, and other entry inhibitors, and is active against CCR5-, CXCR4- and dual-tropic strains of HIV-1 and against *in vitro*–generated CD4-independent HIV-1 (9, 13–15). Analysis of clinical isolates of HIV-1 from fostemsavir-naive patients using the PhenoSense Entry assay (Monogram Biosciences, South San Francisco, CA) has shown a wide range of susceptibilities to temsavir, with most subtypes having 50% inhibitory concentrations (IC_50_s) ranging from <1 nM to >5 μM (14). However, the majority of viruses were considered highly susceptible to temsavir, with only ~9% of virus envelopes exhibiting IC_50_s >100 nM (although CRF01_AE envelopes, which show inherent reduced susceptibility to temsavir were under-represented in this cohort) (14). Data from preclinical studies with temsavir and related experimental attachment inhibitors (BMS-378806 and BMS-488043) and phase 2 clinical studies of fostemsavir indicate that amino acid positions S375, M426, M434, and M475 in HIV-1 gp120 can be relevant in determining phenotypic susceptibility to temsavir and/or reduced virologic response to fostemsavir (16–19).

BRIGHTE (NCT02362503) is an ongoing phase 3 study investigating the efficacy and safety of fostemsavir plus optimized background therapy (OBT) in HTE individuals with confirmed HIV-1 RNA ≥400 copies/mL on their current failing regimen (7, 20). The study comprises a Randomized Cohort of participants with at least 1 but no more than 2 fully-active and approved antiretroviral agents that could be used with fostemsavir, and a Non-randomized Cohort of participants with zero remaining fully active and approved antiretroviral options. At study baseline, both cohorts had high rates of previous exposure across available antiretroviral classes and a correspondingly high frequency of resistance to antiretroviral agents in multiple classes (20). Rates of virologic response (HIV-1 RNA <40 copies/mL) by Snapshot analysis increased between Week 24 and Week 96 (53% [144/272] and 60% [163/272], respectively) in the Randomized Cohort and remained unchanged in the Non-randomized Cohort (37% [37/99] at both time points). There were also clinically relevant mean increases in CD4^+^ T-cell count: +205 and +119 cells/mm^3^ in the Randomized and Non-randomized Cohorts, respectively, at Week 96. Across both cohorts, fostemsavir plus OBT was well tolerated with no new safety signals and few adverse events leading to discontinuation.

In this report, we describe the results from genotypic and phenotypic analyses of baseline and on-treatment samples from participants meeting protocol-defined virologic failure (PDVF) criteria through Week 96 in the BRIGHTE study, with the aim of understanding the contribution to PDVF of polymorphisms at positions of interest in gp120 and changes in viral susceptibility to temsavir and to antiretroviral drugs in the OBT.

## RESULTS

### Baseline characteristics of the PDVF population.

Overall, 371 participants were enrolled in BRIGHTE and received ≥1 dose of study drug: 272 in the Randomized Cohort and 99 in the Non-randomized Cohort. Through Weeks 24, 48, and 96, rates of PDVF were 11% (31/272), 18% (49/272), and 23% (63/272), respectively, in the Randomized Cohort and 28% (28/99), 46% (46/99), and 49% (49/99), respectively, in the Non-randomized Cohort. Baseline characteristics for the Week 96 PDVF population were similar to those for the overall population, which have been previously described, with the exception that the PDVF population had a lower median CD4^+^ T-cell count (Table 1) (7, 21).

**TABLE 1:** Baseline characteristics of the ITT-E and PDVF populations^*a*^

Cohort	Randomized Cohort			Non-randomized Cohort		
Population	ITT-E N = 272	PDVF N = 63	PDVF/ ITT-E	ITT-E N = 99	PDVF N = 49	PDVF/ ITT-E
Parameter	*n* (%)	*n* (%)	%	*n* (%)	*n* (%)	%
Age, yrs						
Median (range)	48 (18-73)	45 (18-66)	NA	50 (17-72)	51 (17-64)	NA
<50	162 (60)	43 (68)	27	44 (44)	19 (39)	43
≥50	110 (40)	20 (32)	18	55 (56)	30 (61)	55
Sex						
Female	72 (26)	15 (24)	21	10 (10)	5 (10)	50
Male	200 (74)	48 (76)	24	89 (90)	44 (90)	49
Race						
White	185 (68)	42 (67)	23	74 (75)	38 (78)	51
Black/African American	60 (22)	13 (21)	22	23 (23)	11 (22)	48
Ethnicity						
Hispanic/Latinx	79 (29)	17 (27)	22	28 (28)	15 (31)	54
HIV-1 RNA, log_10_ c/mL						
Median (range)	4.7 (1.6-6.9)	4.9 (1.6-7.0)	NA	4.3 (1.6-6.6)	4.5 (1.6-6.6)	NA
CD4+ T cells/mm^3^						
Median (range)	99 (0-1160)	60 (0-820)	NA	41 (0-641)	19 (0-346)	NA
Initial OBT FAAs^*b*^						
0 ARV agents	16 (6)^*c*^	4 (6)	25	80 (81)	42 (86)	53
1 ARV agent	142 (52)	30 (48)	21	19 (19)^*d*^	7 (14)	37
2 ARV agents	114 (42)	29 (46)	25	0	NA	NA
>2 ARV agents	0	0	NA	0	NA	NA
Initial OBT OSS						
0	0	0	NA	8 (8)	8 (16)	100
>0-1	27 (10)	9 (14)	33	36 (36)	20 (41)	56
>1-2	131 (48)	30 (48)	23	26 (26)	11 (22)	42
>2	99 (36)	23 (37)	23	26 (26)	9 (18)	35
Missing	15 (6)	1 (2)	7	3 (3)	1 (2)	33
Initial OBT OSS-new						
0	35 (13)	21 (33)	60	55 (56)	33 (67)	60
>0-1	105 (39)	27 (43)	26	30 (30)	14 (29)	47
>1-2	101 (37)	14 (22)	14	12 (12)	2 (4)	17
>2	17 (6)	0	0	1 (1)	0	0
Missing	14 (5)	1 (2)	7	1 (1)	0	0

a ARV, antiretroviral; FAA, fully active ARV; ITT-E, intention-to-treat–exposed; NA, not applicable; OBT, optimized background therapy; OSS, overall susceptibility score; PDVF, protocol-defined virologic failure.

b Including investigational ARVs.

c These included participants who (1) discontinued from the study during the double-blind period and never initiated OBT; (2) had no FAA available at screening and were incorrectly assigned to the Randomized Cohort; or (3) had one or more FAAs available at screening but did not use these as part of the initial OBT.

d 15 of these 19 participants received the investigational ARV ibalizumab and 4 received an approved ARV (2 enfuvirtide, 1 etravirine, and 1 dolutegravir) and were classified as protocol deviations.

The baseline prevalence of predefined amino acid polymorphisms in HIV-1 gp120 (S375H/I/M/N/T, M426L/P, M434I/K, and M475I) was similar between cohorts and between the intention-to-treat–exposed (ITT-E) population and the PDVF population (Table 2 and Table S1). Other amino acid polymorphisms relative to HIV-1 HXB2 at the 4 amino acid positions of interest that were observed at baseline included S375Y, M426I/V/K/R/T, M434T/V, and M475V (Table S1). When these polymorphisms were introduced by site-directed mutagenesis (SDM) into an HIV-1 LAI background, only S375Y (present as a mixture in 2 participants) resulted in a substantial reduction (>10,000-fold) in susceptibility to temsavir. The distribution of baseline temsavir 50% inhibitory concentration fold change (IC_50_ FC) was also similar between cohorts and between the ITT-E population and the PDVF population (Table 2).

**TABLE 2:** Baseline gp120 polymorphisms and temsavir susceptibility for ITT-E and PDVF populations^*a*^

Cohort	Randomized Cohort			Non-randomized Cohort		
Population	ITT-E N = 272	PDVF N = 63	PDVF/ ITT-E	ITT-E N = 99	PDVF N = 49	PDVF/ ITT-E
**Parameter**	*n* (%)	*n* (%)	%	*n* (%)	*n* (%)	%
Baseline gp120 polymorphisms						
Participants with data	**263**	**62**	**24**	**95**	**47**	**49**
No predefined amino acidpolymorphism of interest^*b*^	141 (54)	31 (49)	22	55 (58)	26 (47)	47
At least 1 predefined amino acidpolymorphism^*b*^	122 (46)	31 (49)	25	40 (42)	21 (43)	53
S375H/I/M/N/T	86 (33)	21 (33)	24	25 (26)	12 (24)	48
M426L	32 (12)	11 (17)	34	14 (15)	8 (16)	57
M434I	17 (6)	4 (6)	24	2 (2)	0	0
M475I	3 (1)	0	0	1 (1)	1 (100)	100
More than 1 predefined amino acidpolymorphism^*b*^	16 (6)	5 (8)	31	2 (2)	0	0
Baseline temsavir IC_50_ FC						
Participants with data	**263**	**62**	**24**	**96**	**48**	**50**
≤1	132 (50)	29 (46)	22	49 (51)	22 (45)	45
>1 to 10	63 (24)	14 (22)	22	26 (27)	12 (24)	46
>10 to 100	34 (13)	9 (14)	26	9 (9)	4 (8)	44
>100 to 1000	16 (6)	4 (6)	25	7 (7)	6 (12)	86
>1000	18 (7)	6 (10)	33	5 (5)	4 (8)	80
Geometric mean	2.70	4.12	NA	2.37	4.36	NA
Median (range)	0.99 (0.05-6651.28)	1.38 (0.11-6651.29)	NA	0.94 (0.04-9398.14)	1.49 (0.14-9398.15)	NA

a FC, fold-change in IC_50_ for the test sample relative to a reference control virus; IC_50_, 50% inhibitory concentration; ITT-E, intention-to-treat–exposed; NA, not applicable; PDVF, protocol-defined virologic failure; bold-face numbers are the numbers of participants with data for each sub-heading, and are the denominators that were used to calculate the percentages that are shown in parentheses.

b Predefined amino acid polymorphisms of interest include S375H/I/M/N/T, M426L, M434I, and M475I; M426P and M434K were not present in this study population at baseline. Numbers include mixtures.

In the Randomized Cohort, the initial OBT included 1 fully active antiretroviral by screening criteria for 142 (52%) participants and 2 fully active antiretrovirals for 114 (42%) participants (Table 1). Incidence of PDVF was similar in both these groups. In the Non-randomized Cohort, the initial OBT included zero fully active antiretrovirals for 80 (81%) participants (Table 1). Most of the remaining Non-randomized participants (15/19) included ibalizumab in their initial OBT. Among these 15 participants, rates of PDVF were 13% (2/15), 27% (4/15), and 33% (5/15) through Weeks 24, 48, and 96, respectively.

For both cohorts, there was a broad range of OBT susceptibility scores at baseline and a clear trend toward reduced incidence of PDVF among participants with higher overall susceptibility score to newly used antiretrovirals (OSS-new) (Table 1). In the Randomized Cohort, 239/272 (88%) participants included an integrase inhibitor (INI) and 158/272 (58%) included a protease inhibitor (PI) in the initial OBT. In the Non-randomized Cohort, 75/99 (76%) participants included an INI and 85/99 (86%) included a PI in the initial OBT. Overall, the most common components of the initial OBT were dolutegravir and darunavir (both with twice-daily dosing in most participants); however, in many cases, Monogram susceptibility assays suggested that these antiretrovirals were not fully active at baseline (overall susceptibility ratio [OSR] or overall susceptibility ratio to newly used antiretrovirals [OSR-new] ≠ 1; Table S2). For participants who had an OSR-new = 1 (i.e., suggestive of a fully retained antiretroviral agent) for dolutegravir or darunavir in the OBT, the incidence of PDVF was lower compared with participants for whom dolutegravir or darunavir were either absent, recycled, or otherwise not fully active (Table S2). In the Randomized Cohort, PDVF was met by 25/167 (15%) participants who included dolutegravir with an OSR-new of 1 in their initial OBT and 0/31 participants who included darunavir with an OSR-new of 1 in their initial OBT. Notably, among the 31 participants who included darunavir with an OSR-new of 1 in their initial OBT, the majority also included dolutegravir with an OSR-new of 1 (20/31 [65%]), and 28/31 (90%) had an OSS-new ≥2 for their initial OBT. In contrast, among 167 participants who included dolutegravir with an OSR-new of 1 in their initial OBT, a minority (20/167 [12%]) also included darunavir with an OSR-new of 1, and 86/167 (51%) had an OSS-new ≥2 for their initial OBT.

### Treatment-emergent genotypic and phenotypic changes (Week 96 PDVF).

Of 112 participants with PDVF through Week 96, 107 had resistance testing within the PDVF window. Most participants in the Randomized (54/59 [92%]) and Non-Randomized Cohorts (42/48 [88%]) had resistance testing data available at the time of PDVF, and 2/59 (3%) in the Randomized Cohort had resistance testing from the time of suspected virologic failure. In the Randomized and Non-Randomized Cohorts, 3/59 (5%; range 18–81 days after PDVF) and 6/48 (13%; range 19–120 days), respectively, had resistance testing subsequent to PDVF, during which time they remained on their same treatment regimen. Of 112 participants with PDVF through Week 96, 94 (84%) had HIV-1 gp160 sequence data and 98 (88%) had phenotypic HIV-1 temsavir susceptibility data at baseline and on treatment (Tables 3 and 4). Among those with available HIV-1 gp160 sequence data at both time points, 24/50 (48%) in the Randomized Cohort and 33/44 (75%) in the Non-randomized Cohort had at least 1 treatment-emergent predefined amino acid substitution in gp120 (including 15/50 [30%] in the Randomized Cohort and 14/44 [32%] in the Non-randomized Cohort with multiple emergent substitutions). The most frequent emergent predefined amino acid substitutions were M426L (40%, including M426M/L), S375N (31%, including S375N/T, S375S/N, and S375H/N), M475I (12%, including M475M/I), and M434I (10%, including M434M/I and M434M/I/T; Table 3). Treatment-emergent S375N (with or without other predefined amino acid substitutions, *n* = 14) was associated with temsavir IC_50_ FC at PDVF ranging from 3.3 to 5,784 and a change from baseline to PDVF in temsavir IC_50_ FC ranging from 3.3- to 14,345-fold, and treatment-emergent M426L (with or without other predefined amino acid substitutions, *n* = 23) was associated with temsavir IC_50_ FC at PDVF of 0.4 to 6,651 and a change from baseline to PDVF in temsavir IC_50_ FC from 0.8- to 29,150-fold. Five participants (4 in the Randomized Cohort and 1 in the Non-randomized Cohort) had treatment-emergent S375N plus M426L, associated with temsavir IC_50_ FC ranging from 3.3 to 5,472 and a change from baseline to PDVF in temsavir IC_50_ FC ranging from 3.3 to 12,146.

**TABLE 3:** Treatment-emergent genotypic changes among participants meeting PDVF criteria at Week 96^*a*^

Cohort	Randomized Cohort (N = 272)	Non-randomized Cohort (N = 99)
Participants meeting PDVF, *n* (%)	63 (23)	49 (49)
gp160 sequenced, *n*^*b*^	50	44
Treatment-emergent predefined amino acidsubstitutions in gp120, *n* (%)^*c*^		
None	26 (52)	11 (25)
Any	24 (48)	33 (75)
S375H/I/M/N/T	15 (30)	22 (50)
S375H	0	1 (2)
S375H/N	1 (2)^*d*^	1 (2)
S375M	0	3 (7)
S375N	7 (14)	8 (18)
S375N/T	1 (2)	2 (5)
S375S/I	0	1 (2)
S375S/M/T	1 (2)	0
S375S/N	4 (8)	6 (14)
S375S/T	1 (2)	0
M426L	16 (32)	21 (48)
M426L	10 (20)	13 (30)
M426M/L	7 (14)	8 (18)
M434I	5 (10)	4 (9)
M434I	1 (2)	1 (2)
M434M/I	4 (8)	3 (7)
M434M/I/T	1 (2)	0
M475I	6 (12)	5 (11)
M475I	4 (8)	1 (2)
M475M/I	2 (4)	4 (9)

a PDVF, protocol-defined virologic failure.

b Most missing genotypic data were the result of assay failure, usually because of low HIV-1 RNA levels.

c Predefined amino acid substitutions in gp120 are S375H/I/M/N/T, M426L/P, M434I/K, and M475I; M426P and M434K were not present in any baseline or on-treatment samples from this study population. Numbers include mixtures. The denominator is the number of participants with gp120 sequenced at baseline and on treatment. For each participant, results at additional on-treatment time points around the time of PDVF are included where available (not limited to only the PDVF time point).

d Only S375H was emergent; S375N was present at baseline.

**TABLE 4:** Treatment-emergent changes in temsavir susceptibility among participants meeting PDVF criteria at Week 96^*a*^

Cohort Parameter	Randomized Cohort (N = 272)		Non-randomized Cohort (N = 99)	
Participants meeting PDVF, *n* (%)	63 (23)		49 (49)	
Participants with BL and on-treatment^*a*^ phenotypic data, *n*	53		45	
TMR IC_50_ FC at failure				
Median (range)	142 (0.1-6651)		3320 (0.2-5784)	
Change from BL in TMR IC_50_ FC^*c*^				
≤3-fold	29 (55)		13 (29)	
Median (range)	1.7-fold (0.2-29,150)		470-fold (0.1-24,124)	
Participants with BL and on-treatment^*b*^ genotypic and phenotypic data, *n*	50		44	
Treatment-emergent predefined amino acid substitutions in gp120^*d*^	with (*n* = 24)^*e*^	without (*n* = 26)	with (*n* = 32)^*f*^	without (*n* = 11)
TMR IC_50_ FC at failure, *n* (%)^*g*^				
≤1	2 (8)	12 (46)	1 (3)	1 (9)
>1 to 10	1 (4)	3 (12)	2 (6)	2 (18)
>10 to 100	2 (8)	4 (15)	2 (6)	1 (9)
>100 to 1000	5 (21)	4 (15)	5 (16)	2 (18)
>1000 to 5000	10 (42)	3 (12)	14 (44)	2 (18)
>5000	4 (17)	0	8 (25)	3 (27)
Median (range)	1448 (0.4-6651)	3.1 (0.1-4270)	4279 (0.2-5784)	402 (0.2-5670)
Change from BL in TMR IC_50_ FC, *n* (%)^*g*^				
≤3-fold	3 (13)	23 (88)	3 (9)	9 (82)
>3- to 10-fold	3 (13)	2 (8)	1 (3)	2 (18)
>10- to 100-fold	1 (4)	0	4 (13)	0
>100- to 3000-fold	9 (38)	1 (4)	12 (38)	0
>3000-fold	8 (33)	0	12 (38)	0
Median (range)	511-fold (0.6-29,150)	0.9-fold (0.4-107)	2260-fold (0.1-24,124)	0.7-fold (0.5-5)

a Data are n (%) unless stated otherwise. BL, baseline; PDVF, protocol-defined virologic failure; TMR IC_50_ FC, temsavir 50% inhibitory concentration fold-change.

b On-treatment resistance testing data are shown at the time of confirmed PDVF where available, or the time of the suspected PDVF or a time point nearest, but subsequent to the PDVF time point.

c The denominator is the number of participants with baseline and on-treatment phenotypic data.

d Predefined amino acid substitutions in gp120 are S375H/I/M/N/T, M426L/P, M434I/K, and M475I; M426P and M434K were not present in any baseline or on-treatment samples from this study population.

e Including 15 participants with >1 emergent predefined amino acid substitution in gp120. For these participants, the median (range) TMR IC_50_ FC at failure was 1578 (0.4-6651) and the median (range) change from BL in TMR IC_50_ FC was 718-fold (0.83-14,345).

f Including 14 participants with >1 emergent predefined amino acid substitution in gp120. For these participants, the median (range) TMR IC_50_ FC at failure was 4342 (0.17-5670) and the median (range) change from BL in TMR IC_50_ FC was 4724-fold (0.74-24,124).

g The denominator is the number of participants with baseline and on-treatment genotypic and phenotypic data.

Overall, as expected, temsavir IC_50_ FC tended to be higher for participants in whom treatment-emergent gp120 substitutions of interest had been detected than in those in whom no treatment-emergent substitutions were detected (Table 4). Among Randomized Cohort participants with treatment-emergent gp120 substitutions at failure, the median temsavir IC_50_ FC was 1,448, an increase of 511-fold above baseline IC_50_ FC, compared with median IC_50_ FC of 3.1 and increase of 0.9-fold, respectively, for participants without treatment-emergent gp120 substitutions. In the Non-randomized Cohort, among participants with treatment-emergent gp120 substitutions around the time of failure, the median temsavir IC_50_ FC was 4,279, an increase of 2,260-fold above baseline IC_50_ FC, compared with 402 and increase of 0.7-fold, respectively, for participants without treatment-emergent gp120 substitutions. Among all participants with PDVF and available phenotypic HIV-1 temsavir susceptibility data, 29/53 (55%) in the Randomized Cohort and 13/45 (29%) in the Non-randomized Cohort had a change from baseline to PDVF in temsavir IC_50_ FC that was within the variability of the phenotypic assay (≤3-fold; Table 4).

### Treatment-emergent resistance to OBT.

**FIG 1:**
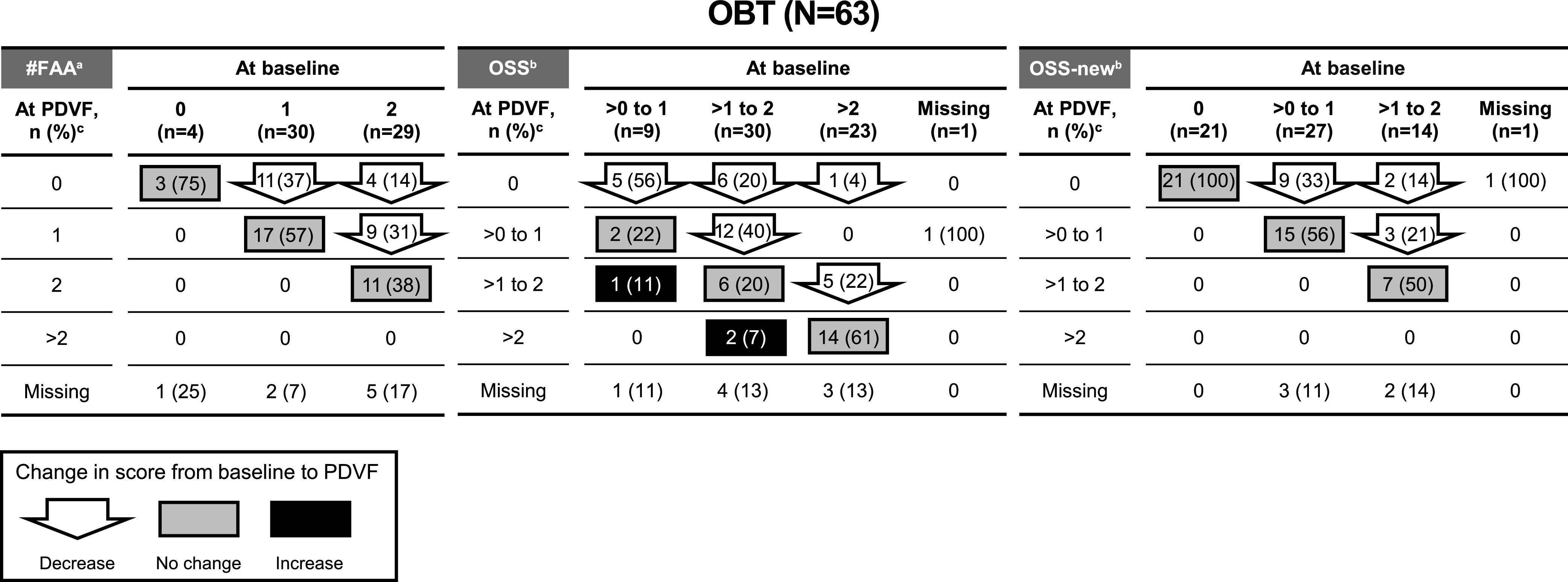
Changes in the distribution of overall susceptibility scores for the initial optimized background therapy from baseline to PDVF among Randomized Cohort participants with PDVF through Week 96 (N = 63). #FAA, number of fully active antiretrovirals; OSS, overall susceptibility score; PDVF, protocol-defined virologic failure. ^a^Full activity was based on susceptibility according to current or historical resistance measures and availability (tolerance, eligibility, and in the case of enfuvirtide only, willingness to take the antiretroviral agent). ^b^Susceptibility scores were based on Monogram susceptibility assays. For OSS-new, only antiretroviral agents not previously used by the participant were scored. If resistance testing results were unavailable for individual components of the initial OBT, this impacted the number of participants included in each population (#FAA, OSS, and OSS-new) differently, depending on whether the agent with missing data was a fully active agent, a new agent, or neither. ^c^PDVF susceptibility data are selected from first of confirmed PDVF date, suspected PDVF (sentinel) date, or first date within 6 months after sentinel date. Percentages reported are based on the sample size of each baseline value.

For all participants meeting PDVF through Week 96, an assessment of emergent resistance to the initial OBT was performed by evaluating the number of fully active antiretrovirals (#FAA; Randomized Cohort only), overall susceptibility score (OSS), and OSS-new of the initial OBT at both baseline and PDVF (Fig. 1). In the Randomized Cohort, among the 55 participants with PDVF through Week 96 and both baseline and PDVF #FAA for their initial OBT, 24 (44%) had a decrease in #FAA at PDVF, reflecting emergent resistance to one or more components of the OBT and 31 (56%) had no change in #FAA, indicative of no emergence of resistance to components of the OBT. Among the 54 participants with PDVF through Week 96 and both baseline and PDVF OSS for their initial OBT, 29 (54%) showed a decrease in OSS at PDVF and 22 (41%) had an OSS that was unchanged. Unchanged OSS from baseline to PDVF was more frequent among participants with baseline OSS >2 (14/23 [61%]) than among those with baseline OSS >0 to 2 (8/39 [21%]). For OSS-new, 14/57 (25%) showed a decrease at PDVF and 43/57 (64%) remained unchanged. Median baseline temsavir IC_50_ FC values were similar in Randomized Cohort participants with or without a decrease in #FAA, OSS, or OSS-new categories from baseline to PDVF (Table S3). Among participants with PDVF and both baseline and PDVF #FAA, OSS, or OSS-new scores for their initial OBT, 14/55 (25%), 12/54 (22%), and 17/57 (30%), respectively, had no change in the OBT susceptibility score and no detectable change in temsavir susceptibility (emergent gp160 substitutions of interest or a >3-fold change in temsavir IC_50_ FC from baseline to PDVF). In the Non-randomized Cohort, OSS and OSS-new remained unchanged for 25/39 (64%) participants and 38/48 (79%) participants, respectively (Fig. S1).

**FIG 2:**
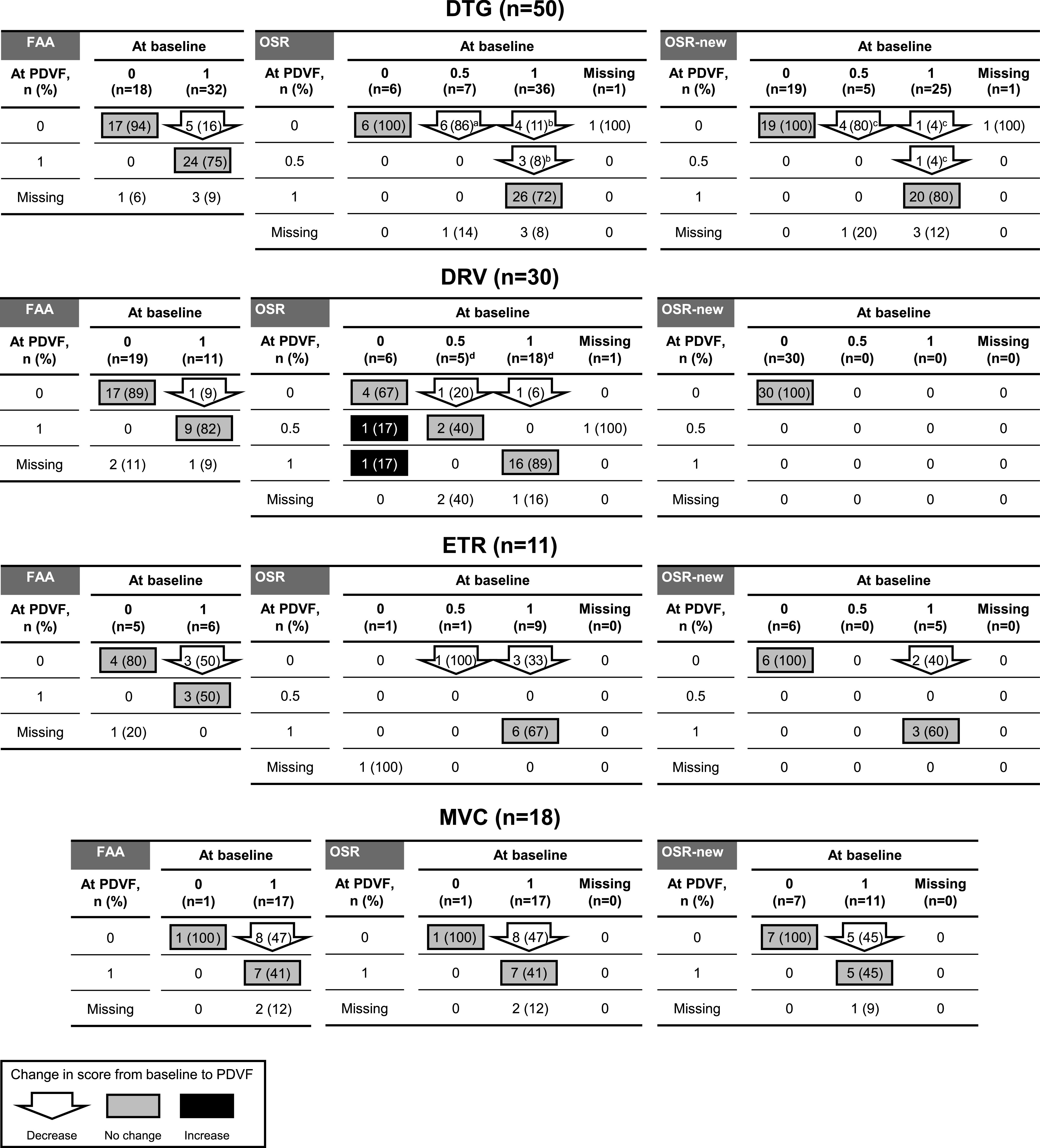
Changes in the distribution of overall susceptibility ratings for common components of the initial optimized background therapy from baseline to PDVF among Randomized Cohort participants with PDVF through Week 96 (N = 63). Participants with missing data at baseline or PDVF are not shown. Percentages reported are based on the sample size of each baseline value. FAA, fully active antiretroviral; DRV, darunavir; DTG, dolutegravir; ETR, etravirine; MVC, maraviroc; OSR, overall susceptibility rating; PDVF, protocol-defined virologic failure; RAL, raltegravir. ^a^All 6 had previous integrase inhibitor treatment experience, 6/6 with RAL, 2/6 with DTG. ^b^All 7 had previous integrase inhibitor treatment experience, 1/7 with DTG, 2/7 with RAL, 4/7 with DTG + RAL. ^c^All 6 had previous integrase inhibitor treatment experience, 6/6 with RAL. ^d^All 23 had previous DRV treatment experience.

A similar assessment was carried out on changes in susceptibility to commonly used individual antiretrovirals in the initial OBT evaluating full activity by inclusion criteria fully active antiretrovirals (FAA), OSR, and OSR-new at baseline and PDVF (Fig. 2 and Fig. S2). In the Randomized Cohort, for most participants in the PDVF population who had included fully active dolutegravir or darunavir in their initial OBT, these agents remained fully active at PDVF (24/32 [75%] and 9/11 [82%]; Fig. 2). All 13 participants in whom the OSR for dolutegravir decreased between baseline and PDVF had previously received an INI and 7 (54%) had previously received dolutegravir. Among 25 participants with an OSR-new of 1 for dolutegravir at baseline, only 2 had a reduction in dolutegravir OSR-new at PDVF and both had previously received raltegravir. In the Non-randomized Cohort (Fig. S2), OSR or OSR-new scores of 1 at baseline were infrequent and were more likely to have shifted to 0 at PDVF.

Samples from PDVF were available for 4 of the 5 participants in the Non-randomized Cohort who experienced virologic failure with an OBT that included ibalizumab. All 4 exhibited decreased susceptibility to ibalizumab and 3 also had decreased susceptibility to temsavir and emergent substitutions of interest in gp120 (M426L or M475I, with S375T, H, or N). Back mutation of codons 375, 426, and 475 to wild-type in individual clones from these 3 PDVF samples resulted in restoration of susceptibility to temsavir but not to ibalizumab, indicating that the reduced susceptibility to temsavir and ibalizumab was not linked (15). Of note, 3 of the 4 participants also had 1 or 2 fewer N-linked glycosylation sites in the V5 region in their PDVF samples compared with their screening samples (15), which is indicative of decreased susceptibility to ibalizumab (22, 23). The remaining participant exhibited reduced susceptibility to ibalizumab at PDVF compared with the screening sample, although the V5 regions at the 2 time points were identical. Thus, the correlates for reduced susceptibility to ibalizumab in this participant are unknown.

### Virologic response after PDVF.

Of participants who experienced PDVF through Week 96, 22 subsequently achieved HIV-1 RNA <40 copies/mL at a later time point before the date of the Week 96 data lock (August 2018) while still receiving fostemsavir: 17/63 (27%) in the Randomized Cohort and 5/49 (10%) in the Non-randomized Cohort. In the Randomized Cohort, among 12 participants who had evaluable gp120 genotypic data at PDVF, 7 (58%) achieved virologic suppression after PDVF without any change in OBT, and in 2 of these 7 individuals, there was emergence of gp120 amino acid substitutions of interest and a >100-fold increase in temsavir IC_50_ FC from baseline to PDVF. Of the 5/12 remaining participants who experienced virologic suppression post-PDVF with a change made to OBT, 1 had a change in OBT before PDVF and 4 had a change in OBT at or after the time of PDVF. In 3 of these cases, virologic suppression was achieved with treatment-emergent gp120 amino acid substitutions of interest and a >100-fold increase in temsavir IC_50_ FC from baseline to PDVF. In the Non-randomized Cohort, 3 (60%) of the 5 participants who achieved virologic suppression after PDVF did so with no change to OBT. In 2 of these 3 individuals, there was emergence of gp120 amino acid substitutions of interest and a >100-fold increase in temsavir IC_50_ FC at PDVF.

## DISCUSSION

In the Randomized Cohort of the ongoing BRIGHTE study, the rate of PDVF through Week 96 (23%) was not unexpected considering the extensive previous antiretroviral experience of the study population and was similar to rates previously reported for other populations with multidrug-resistant virus (24, 25). The incidence of PDVF through Week 96 was similar among Randomized Cohort participants with either 1 or 2 fully active antiretroviral agents included as part of their initial OBT (26). This may reflect the fact that the most commonly used fully active antiretroviral agent in the initial OBT was dolutegravir (27), which is an effective backbone agent with a high barrier to resistance and proven efficacy in treatment-experienced people living with multidrug-resistant HIV-1 (28–32). Thus, the activity of another fully active antiretroviral in the OBT may not have resulted in a measurable increase in response. The incidence of PDVF in participants who included dolutegravir in the initial OBT was lower relative to those without dolutegravir in the initial OBT, particularly where dolutegravir was fully active (OSR or OSR-new of 1).

The higher incidence of PDVF in the Non-randomized Cohort (49%) was consistent with the lack of approved fully active antiretroviral agents available for these participants to include in their OBT (67% had an OSS-new of 0 for the initial OBT) and their more advanced disease status (indicated by lower baseline CD4^+^ T-cell counts compared with the Randomized Cohort). In clinical practice, individuals with no remaining fully active agents available from currently approved antiretroviral classes may reduce the risk of virologic failure by including new classes of antiretrovirals in their treatment regimen (through enrollment in clinical trials or expanded access programs).

Notably, baseline CD4^+^ T-cell counts were lower among participants with PDVF compared with the overall population for both cohorts, further implicating advanced disease as a risk factor for virologic failure. Conversely, in spite of more advanced HIV disease and broader multidrug antiretroviral resistance in the Non-randomized Cohort (7, 20), at baseline, the distribution of predefined amino acid substitutions in HIV-1 gp120 and the levels of temsavir susceptibility were similar between the Randomized and Non-randomized Cohorts (Table 2). This is consistent with the previously described lack of cross-resistance between temsavir and other antiretrovirals (9, 13) and suggests that a longer duration of prior antiretroviral treatment, as observed in the Non-randomized Cohort, is unlikely to lead to selection of changes in gp120 that may impact response to fostemsavir. This observation is further supported by an analysis of 23 patients in the Italian PRESTIGIO registry with 23 to 27 years of antiretroviral treatment experience; samples from all 23 had temsavir susceptibility comparable to the NL4-3 or AD8 controls (33).

The specific gp120 amino acid substitutions S375H/I/M/N/T, M426L/P, M434I/K, and M475I (HXB2 numbering) were predefined for evaluation in this analysis on the basis of previous *in vitro* and *in vivo* evidence of their association with reduced susceptibility to temsavir (14–17). Other changes at the 4 amino acid positions of interest, such as M426I/T/R, are considered to have a neutral effect on temsavir IC_50_ FC and were not included in the analysis (9, 15, 16). The prevalence of predefined gp120 amino acid substitutions in the overall study population was similar to their prevalence among sequences in the 2019 LANL HIV Sequence Database (34), indicative of a lack of selective pressure at these positions in fostemsavir-naive, multidrug-resistant viral envelopes. In BRIGHTE, the most prevalent amino acid substitution at baseline was S375T (15%). Previous *in vitro* analyses have shown that the impact of S375T on viral susceptibility to temsavir is relatively minor compared with some of the other polymorphisms of interest at this position and other positions (17). Although S375Y, found as a part of a mixed virus population in 2 BRIGHTE study participants, was shown to result in a large reduction in susceptibility to temsavir *in vitro*, this polymorphism is extremely rare across all HIV-1 subtypes (https://www.hiv.lanl.gov/content/sequence/HIV/mainpage.html), which may be indicative of a lack of viral fitness at the population level, and was not observed during *in vitro* passage experiments with temsavir (17).

Among participants with PDVF, no specific baseline or on-treatment pattern in genotypic or phenotypic temsavir susceptibility was identified; however, approximately half had one or more gp120 substitutions of interest that emerged during treatment. Consistent with previous observations (16–18), these were generally associated with increases in temsavir IC_50_ FC from baseline to PDVF, although there was a wide range of temsavir IC_50_ FC seen in clinical samples with specific substitutions. Correlation of emergent substitutions with temsavir IC_50_ FC is confounded by the presence of mixtures and the emergence of multiple substitutions. Even allowing for the fact that not all substitutions have an equal influence on temsavir IC_50_ FC, the data suggest that the impact of these changes may depend on genetic context (i.e., on the amino acid sequence of the full gp120). Conversely, at failure, 52% of participants with PDVF in the Randomized Cohort and 25% in the Non-randomized Cohort had no treatment-emergent gp120 substitutions of interest, and 55% and 29% in the Randomized and Non-randomized Cohorts, respectively, had a change in baseline temsavir IC_50_ FC within the variability of the assay (≤3-fold). Therefore, gp120 polymorphisms and treatment-emergent gp120 substitutions of interest and phenotypic changes in temsavir susceptibility could not account for all cases of PDVF in this study. An exploratory analysis of the Randomized Cohort Day 8 Virologic Outcome population found a weak positive relationship between baseline temsavir IC_50_ FC and virologic response, with substantial variability, further supporting that temsavir phenotypic susceptibility is not a reliable predictor of PDVF in this study (35).

In the absence of a clear association between PDVF and reduced susceptibility to temsavir, other factors must be considered. In BRIGHTE, both OSS and OSS-new were evaluated as alternative ways to identify factors that might be most closely related to clinical outcome. Results from the VIKING-3 study (36) showed that when drugs previously used were excluded from the calculations, the summed susceptibility score of the OBT decreased substantially and an association between this score and virologic response rate was more apparent, indicating that using the summed susceptibility score of newly used agents only provided a better reflection of the antiviral activity of the OBT. In the Randomized Cohort, there was no clear relationship between OSS of the initial OBT and incidence of PDVF, although there was a trend toward reduced incidence of PDVF among participants with higher OSS-new, indicating that OSS-new may be a more reliable predictor of virologic outcome in HTE individuals.

The OSS of the initial OBT in this study was based on the results of resistance testing at screening only and may have overestimated the baseline activity of some agents. The decreases in OSS from baseline to time of failure, observed in 29/54 (54%) of Randomized Cohort participants with PDVF, may reflect archived resistance that has re-emerged under selective pressure from antiretroviral agents being recycled in the initial OBT. For OSS-new, the score decreased from baseline to time of failure in only 14/57 (25%) Randomized Cohort participants, suggesting that this score may provide a better measure for predicting the baseline antiviral activity of antiretrovirals in the OBT. In the Randomized Cohort, the only antiretroviral class for which most participants had not exhausted all options was the INIs (7). As a result, the most common antiretroviral with an OSR and OSR-new of 1 in the initial OBT of Randomized Cohort participants was dolutegravir (70% and 61%, respectively). Most cases of PDVF among these participants were not associated with any detectable reduction in susceptibility to dolutegravir, suggesting that other factors were the cause of failure. Notably, all participants who experienced a loss of predicted susceptibility to dolutegravir included in their initial OBT (i.e., a reduction in OSR for dolutegravir between baseline and PDVF) had previously been treated with INIs, and some had received prior dolutegravir. It is plausible that fostemsavir may offer additional protection to a partially active background regimen due to its extracellular mechanism of action. Viruses carrying mutations associated with resistance to antiretrovirals that inhibit intercellular steps in the viral life cycle may be prevented from entering uninfected cells, and replicating to become the dominant variants, by the complementary extracellular activity of temsavir.

Participants in the Non-randomized Cohort, who had no remaining fully active antiretrovirals available, included PIs and nucleoside reverse transcriptase inhibitors in their initial OBT more frequently than Randomized Cohort participants (20). Since there was already extensive prior experience with and/or resistance to antiretrovirals in the initial OBT in the Non-randomized Cohort, there was limited potential for further evolution of resistance; therefore, OSS and OSS-new mostly remained unchanged between baseline and PDVF.

Notably, in the Randomized Cohort, approximately one-quarter of participants with PDVF who had available data at both baseline and PDVF had no emergent changes in susceptibility to temsavir and no decrease in susceptibility to the OBT. Virologic failure without emergent genotypic or phenotypic changes to fostemsavir and/or background agents may also be a result of lack of selective drug pressure, which may be indicative of incomplete adherence to the treatment regimen. There are known challenges to optimal antiretroviral adherence among HTE individuals, including pill burden and treatment interruptions resulting from adverse events or comorbidities, supporting the fact that virologic failure may not always warrant a change in the antiretroviral regimen.

Analysis of data from the participants who met criteria for PDVF but later achieved virologic suppression (27% of participants with PDVF in the Randomized Cohort and 10% in the Non-randomized Cohort) revealed a unique profile for each participant with a mixture of factors likely contributing to the observed pattern of virologic response, including variable adherence to treatment and initial choice of and subsequent adjustments to components of the OBT.

To date, no clinical cutoff or genotypic algorithm has been established that can reliably predict clinical efficacy outcomes to fostemsavir-based therapy. The interpretation of data from participants who experienced PDVF in BRIGHTE is complicated by the numerous confounding factors possible in a study population with a wide variety of individualized OBTs, varying degrees of immune suppression, and numerous comorbidities (20). Understanding the contribution of baseline or emergent genotypic changes in gp120 to virologic failure is further confounded by the observation that the impact of individual amino acid substitutions on phenotypic susceptibility to temsavir is highly variable and dependent on the sequence context of the envelope gp120 in which the substitution is found (17–19). In this HTE population with advanced disease, multidrug-resistant HIV-1, and a pressing need for new treatment options, the majority of participants in the Randomized Cohort and more than one-third of participants in the Non-randomized Cohort achieved virologic suppression through Week 96. Among HTE participants in the phase 3 BRIGHTE study who met PDVF criteria through Week 96, we could identify no consistent pattern of emergent changes in susceptibility to temsavir or to components of the OBT. Different combinations of factors are likely to be involved in individual cases of PDVF; further studies will be required to identify which factors are most likely to contribute to virologic failure while receiving an optimized treatment regimen including fostemsavir, and what can be done to mitigate those factors.

## MATERIALS AND METHODS

### Study design and participants.

The BRIGHTE study is a 2-cohort, phase 3, randomized, placebo-controlled, double-blind clinical trial being conducted at 108 centers in 22 countries across Africa, Asia-Pacific, Europe, North America, and South America. The study design has previously been described (7, 20). Participants were enrolled between February 2015 and May 2016. Eligible participants were aged ≥18 years, had plasma HIV-1 RNA ≥400 copies/mL on their current failing antiretroviral regimen, and were unable to form a complete antiretroviral regimen out of remaining fully active and approved agents. Full activity was based on susceptibility (according to current and historical resistance measures) and availability (tolerance, eligibility, and in the case of enfuvirtide only, willingness to take the antiretroviral agent). There was no temsavir IC_50_ criterion for study entry. Full details of inclusion and exclusion criteria are available online at https://www.viiv-studyregister.com/en/study/?id=205888.

Participants in the Randomized Cohort were required to have at least 1, but no more than 2, approved fully active antiretroviral agents remaining that could be combined with fostemsavir as part of a viable antiretroviral regimen. These participants were randomized 3:1 to receive blinded fostemsavir 600 mg or placebo twice daily along with their failing antiretroviral regimen from Day 1 until Day 8. After Day 8, all Randomized Cohort participants received open-label fostemsavir with an individualized OBT regimen chosen at the discretion of the managing investigator. Participants in the Non-randomized Cohort had no remaining approved fully active antiretrovirals. These participants received open-label fostemsavir 600 mg twice daily plus an individualized OBT from Day 1. Participants in the Non-randomized Cohort were permitted to co-enroll in clinical trials being conducted on other investigational antiretroviral agents (eg, ibalizumab, which was not approved at the time of enrollment).

The study was conducted in accordance with international laws and guidelines consistent with the Declaration of Helsinki principles, with oversight from national, regional, or institutional review boards or ethics committees. All study participants provided informed consent. BRIGHTE is expected to continue until participants can access fostemsavir through other means (eg, marketing approval).

### Procedures.

Genotypic and phenotypic resistance testing of isolates was carried out for all participants at screening and at the time of virologic failure, for participants who met PDVF criteria, through Week 96. The criteria for PDVF before Week 24 were confirmed (or last available before discontinuation) HIV-1 RNA ≥400 copies/mL after confirmed suppression to <400 copies/mL or confirmed (or last available before discontinuation) >1 log_10_ copies/mL increase in HIV-1 RNA above nadir where nadir is ≥40 copies/mL. The criteria for PDVF on or after Week 24 were confirmed (or last available before discontinuation) HIV-1 RNA ≥400 copies/mL. In both cases, the confirmation sample was to be taken within 4 weeks of the original sample. On-treatment genotypic and phenotypic data were collected from the PDVF confirmation samples. If samples for resistance testing were not available/analyzable at the PDVF confirmation time point, then the suspected PDVF (sentinel) sample was used. If necessary, a sample collected no more than 6 months after confirmed PDVF could be used.

HIV-1 RNA testing was carried out by central laboratory facilities (Laboratory Corporation of America [Indianapolis, IN, USA; Geneva, Switzerland; The Synergy, Singapore]). Genotypic and phenotypic susceptibility assessments were performed by Monogram Biosciences using the PhenoSense GT plus Integrase, PhenoSense Entry, and Trofile Coreceptor Tropism assays. If the PhenoSense GT plus Integrase failed, then samples were reflex-tested using PhenoSense GT, PhenoSense Integrase, GenoSure PRIme, and GeneSeq Integrase assays. Temsavir phenotypic results from the PhenoSense Entry assay are expressed as the fold change (FC) in IC_50_ for the test sample relative to a reference control pseudotype virus (IC_50_ for the reference control is approximately 1 nM). Assay validation experiments show that >95% of replicate measures of IC_50_ FC fall within a 3-fold range (assay data on file at Monogram Biosciences) (18). Changes in susceptibility to temsavir during treatment were expressed as the ratio of on-treatment IC_50_ FC to baseline IC_50_ FC. Population sequencing of the entire gp160 envelope gene was performed by Monogram Biosciences using their next-generation sequencing platform. The baseline and on-treatment presence of predefined amino acid substitutions in gp120 that have demonstrated the potential to reduce HIV-1 susceptibility to temsavir (S375H/I/M/N/T, M426L/P, M434I/K, and M475I) was assessed (9, 17, 18). Other baseline gp120 amino acid polymorphisms that differ from the subtype B consensus at positions 375, 426, 434, and 475 were also recorded, and the impact of these polymorphisms on susceptibility to temsavir was assessed in the context of the HIV-LAI envelope using SDM and a cell-cell fusion assay as previously described (17, 19).

### Assessments and endpoints.

Genotypic and phenotypic drug susceptibility at baseline and on treatment were assessed as planned secondary endpoints in the Randomized Cohort and as an exploratory endpoint in the Non-randomized Cohort. Results are summarized for the ITT-E population (all participants who received at least 1 dose of study treatment) and the PDVF population. Emergence of genotypic and phenotypic changes relative to baseline were assessed in participants with PDVF, including predefined substitutions in HIV-1 gp120 and phenotypic susceptibility to temsavir.

Ad hoc analyses were carried out to assess changes from baseline to treatment failure in the predicted activity of agents in the initial OBT. This was measured by #FAA according to inclusion criteria and by susceptibility scores based on Monogram genotypic and phenotypic assays as described in section S1 of the supplemental material. To calculate an OSS, each antiretroviral agent in the OBT was assigned an OSR based on the net results from the Monogram assays (1.0 = full activity, 0.5 = partial activity, 0 = reduced susceptibility or resistance), and the OSRs were summed. “OSS-new” was a variation of OSS in which only antiretroviral agents not previously used by the participant were scored (section S1 of the supplemental material).
