# Supplementary material for: Week 96 Genotypic and Phenotypic Results of the Fostemsavir Phase 3 BRIGHTE Study in Heavily Treatment-Experienced Adults Living with Multidrug-Resistant HIV-1
Source: Antimicrob Agents Chemother. 2022 May 3;66(6):e01751-21. doi: 10.1128/aac.01751-21 (PMC9211436; doi:10.1128/aac.01751-21)
Supplement: Supplemental file 1 — Tables S1 to S3, Fig. S1 and S2, and Sections S1 and S2. Download aac.01751-21-s0001.pdf, PDF file, 0.6 MB [file aac.01751-21-s0001.pdf]

SUPPLEMENTAL TABLE S1 Summary of genotypic substitutions at amino acid positions of interest in gp120 at baseline (intention-to-treat–exposed population)

	Substitution, n (%)[a]			
	**Randomized Cohort (N=272)**	**Non-randomized Cohort (N=99)**	**Total (N=371)**	**TMR susceptibility of SDM, FC IC_{50}[b]**
Sequenced	263 (97)	95 (96)	358 (96)	
Any substitution at a position of interest in gp120[c]	157 (60)	55 (58)	212 (59)	
Predefined substitutions of interest in gp120[d]	122 (46)	40 (42)	162 (45)	
S375 any	87 (33)	25 (26)	112 (31)	
S375 predefined (H/I/M/N/T)[e]	86 (33)	25 (26)	111 (31)	
S375H	1 (<1)	1 (1)	2 (<1)	48
S375I	3 (1)	1 (1)	4 (1)	17
S375S/I	1 (<1)	0	1 (<1)	
S375M	3 (1)	4 (4)	7 (2)	47

S375S/M	1 (<1)	0	1 (<1)	
S375I/M	2 (<1)	0	2 (<1)	
S375I/T	1 (<1)	0	1 (<1)	
S375M/T	1 (<1)	0	1 (<1)	
S375N	12 (5)	3 (3)	15 (4)	1
S375S/N	13 (5)	1 (1)	14 (4)	
S375S/N/T	1 (<1)	0	1 (<1)	
S375S/N/Y	1 (<1)	0	1 (<1)	
S375T	39 (15)	15 (16)	54 (15)	1
S375S/T	7 (3)	0	7 (2)	
S375S/Y	1 (<1)	0	1 (<1)	>10,000
M426 any	94 (36)	35 (37)	129 (36)	
M426 predefined (L)[e]	32 (12)	14 (15)	46 (13)	
M426L	29 (11)	10 (11)	39 (11)	98
M426M/L	3 (1)	4 (4)	7 (2)	
M426I/V	1 (<1)	0	1 (<1)	1.8/3.3

M426K	4 (2)	0	4 (1)	0.5
M426M/K	1 (<1)	0	1 (<1)	
M426R	52 (20)	17 (18)	69 (19)	0.86
M426M/R	4 (2)	1 (1)	5 (1)	
M426T	0	1 (1)	1 (1)	0.42
M426M/T	0	2 (1)	2 (1)	
M434 any	23 (9)	2 (2)	25 (7)	
M434 predefined (I)[e]	17 (6)	2 (2)	19 (5)	
M434I	10 (4)	0	10 (3)	2
M434M/I	6 (2)	2 (2)	8 (2)	
M434M/I/V	1 (<1)	0	1 (<1)	
M434T	1 (<1)	0	1 (<1)	15
M434M/T	4 (2)	0	4 (1)	
M434V	1 (<1)	0	1 (<1)	7.5
M475 any	4 (2)	2 (2)	6 (2)	
M475 predefined (I)	3 (1)	1 (1)	4 (1)	

M475I	3 (1)	1 (1)	4 (1)	11
M475V	0	1 (1)	1 (<1)	9.5
M475M/V	1 (<1)	0	1 (<1)	
Predefined substitutions of interest at ≥1 amino acid position in gp120[c,d]				
Including S375H/I/M/N/T	14 (5)	2 (2)	16 (4)	
Including M426L	8 (3)	1 (1)	9 (3)	
Including M434I	8 (3)	1 (1)	9 (3)	
Including M475I	2 (<1)	0	2 (<1)	

FC, fold-change; IC_{50}, 50% inhibitory concentration; SDM, site-directed mutant; TMR, temsavir.

Shaded rows identify amino acid substitutions not included in the predefined substitutions of interest.

[a]Sequenced percentages are based on population count and other percentages are based on participants with sequenced results. [b]Susceptibility of SDMs of the HIV-1 LAI envelope containing observed baseline polymorphisms to temsavir in a cell-cell fusion assay. Results are expressed as IC_{50} for the SDM/IC_{50} for the LAI control. [c]Amino acid positions of interest in gp120 are 375, 426, 434, and 475. [d]Predefined substitutions of interest are S375H/I/M/N/T, M426L/P, M434I/K, and M475I: M426P and M434K were not present in this study population at baseline. [e]Including mixtures.

SUPPLEMENTAL TABLE S2 Contribution of common ARVs in the initial OBT

Cohort	Randomized Cohort			Non-randomized Cohort		
**Population**	**ITT-E N=272**	**PDVF N=63**	**PDVF/ ITT-E**	**ITT-E N=99**	**PDVF N=49**	**PDVF/ ITT-E**
**ARVs in OBT[a]**	**n (%)**	**n**	**%**	**n (%)**	**n**	**%**
**Dolutegravir in OBT**	**229 (84)[b]**	**50**	**22**	**74 (75)[c]**	**37**	**50**
OSR = 1	*190 (70)*	*36*	*19*	*28 (28)*	*9*	*32*
OSR = 0.5	*22 (8.1)*	*7*	*32*	*12 (12)*	*6*	*50*
OSR = 0	*10 (3.7)*	*6*	*60*	*32 (32)*	*21*	*66*
OSR missing	*7 (2.6)*	*1*	*14*	*2 (2.0)*	*1*	*50*
OSR-new = 1	*167 (61)*	*25*	*15*	*20 (20)*	*5*	*25*
OSS-new = 0.5	*16 (5.9)*	*5*	*31*	*7 (7.1)*	*4*	*57*
OSR-new = 0	*39 (14)*	*19*	*49*	*46 (46)*	*28*	*61*
OSR-new missing	*2 (<1)*	*1*	*50*	*0*	*0*	*—*
No dolutegravir in OBT	43 (16)	13	30	25 (25)	12	48
**Darunavir in OBT**	**134 (49)[d]**	**30**	**22**	**71 (72)[e]**	**34**	**48**

OSR = 1	*79 (29)*	*18*	*23*	*14 (14)*	*4*	*29*
OSS = 0.5	*22 (8.1)*	*5*	*23*	*19 (19)*	*9*	*47*
OSR = 0	*31 (11)*	*6*	*19*	*37 (37)*	*21*	*57*
OSR missing	*2 (<1)*	*1*	*50*	*1*	*0*	*0*
OSR-new = 1	*31 (11)*	*0*	*0*	*5 (5.1)*	*0*	*0*
OSS-new = 0.5	*11 (4.0)*	*0*	*0*	*1 (1.0)*	*0*	*0*
OSR-new = 0	*92 (34)*	*30*	*33*	*65 (66)*	*34*	*52*
OSR-new missing	*0*	*0*	*—*	*0*	*0*	*—*
No darunavir in OBT	138 (51)	33	24	28 (28)	15	54
**Etravirine in OBT**	**54 (20)**	**11**	**20**	**21 (21)**	**10**	**48**
OSR = 1	*48 (18)*	*9*	*19*	*7 (7.1)*	*1*	*14*
OSS = 0.5	*1 (<1)*	*1*	*100*	*5 (5.1)*	*4*	*80*
OSR = 0	*5 (1.8)*	*1*	*20*	*9 (9.1)*	*5*	*56*
OSR missing	*1*	*0*	*0*	*0*	*0*	*—*
OSR-new = 1	*39 (14)*	*5*	*13*	*4 (4.0)*	*1*	*25*
OSS-new = 0.5	*0*	*0*	*—*	*3 (3.0)*	*3*	*100*

OSR-new = 0	*15 (5.5)*	*6*	*40*	*14 (14)*	*6*	*43*
OSR-new missing	*1 (<1)*	*0*	*0*	*0*	*0*	*—*
No etravirine in OBT	218 (80)	52	24	78 (79)	39	50
**Maraviroc in OBT**	**52 (19)**	**18**	**35**	**8 (8)**	**4**	**50**
OSR = 1	*49 (18)*	*17*	*35*	*4 (4.0)*	*2*	*50*
OSR = 0	*1 (<1)*	*1*	*100*	*3 (3.0)*	*2*	*67*
OSR missing	*2 (<1)*	*0*	*0*	*1 (1.0)*	*0*	*0*
OSR-new = 1	*36 (13)*	*11*	*31*	*2 (2.0)*	*1*	*50*
OSR-new = 0	*14 (5.1)*	*7*	*50*	*6 (6.1)*	*3*	*50*
OSR-new missing	*2 (<1)*	*0*	*0*	*0*	*0*	*—*
No maraviroc in OBT	220 (81)	45	20	91 (92)	45	49

ARV, antiretroviral; BID, twice daily; ITT-E, intention-to-treat–exposed; NRTI, nucleoside reverse transcriptase inhibitor; OBT, optimized background therapy; OSR, overall susceptibility rating; PDVF, protocol-defined virologic failure.

[a]Including ARVs other than NRTIs that were used in the OBT of more than 15% of the study population. [b]Dosed BID in 171/229 (75%). [c]Dosed BID in 68/74 (92%). [d]Dosed BID in 99/134 (74%). [e]Dosed BID in 63/71 (89%).

SUPPLEMENTAL TABLE S3 Median baseline TMR IC_{50} FC by #FAA, OSS, and OSS-new categories in Randomized Cohort participants meeting PDVF through Week 96 (N=63)

Category change to initial OBT	n[a]	Median baseline TMR IC_{50} FC
With decrease in #FAA	24	1.565
Without decrease in #FAA	30	1.195
With decrease in OSS category	29	1.690
Without decrease in OSS category	24	0.915
With decrease in OSS-new category	14	2.245
Without decrease in OSS-new category	42	1.195

#FAA, number of fully-active antiretrovirals; OBT, optimized background therapy; OSS, overall susceptibility score; PDVF, protocol-defined virologic failure; TMR IC_{50} FC, temsavir 50% inhibitory concentration fold-change.

[a]Excluding participants without baseline TMR IC_{50}.

SUPPLEMENTAL FIG S1. Changes in the distribution of overall susceptibility scores for the initial optimized background therapy from baseline to PDVF among participants in the Non-randomized Cohort with PDVF (N=49). OSS, overall susceptibility score; PDVF, protocol-defined virologic failure.

**OSS**	**At baseline**				
**At PDVF, n (%)**	**0 (n=8)**	**>0 to 1 (n=20)**	**>1 to 2 (n=11)**	**>2 (n=9)**	**Missing (n=1)**
0	5 (63)	7 (35)	0	1 (11)	1 (100)
>0 to 1	2 (25)	10 (50)	2 (18)	0	0
>1 to 2	0	1 (5)	4 (36)	0	0
>2	0	0	1 (9)	6 (67)	0
Missing	1 (13)	2 (10)	4 (36)	2 (22)	0

**OSS-new**	**At baseline**				
**At PDVF, n (%)**	**0 (n=33)**	**>0 to 1 (n=14)**	**>1 to 2 (n=2)**	**>2 (n=0)**	**Missing (n=0)**
0	32 (97)	8 (57)	0	0	0
>0 to 1	0	5 (36)	1 (50)	0	0
>1 to 2	0	1 (7)	1 (50)	0	0
>2	0	0	0	0	0
Missing	1 (3)	0	0	0	0

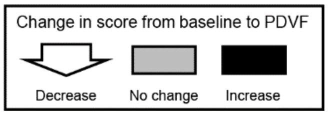

SUPPLEMENTAL FIG S2. Changes in the distribution of overall susceptibility ratings for common components of the initial optimized background therapy from baseline to PDVF among participants with PDVF (Non-randomized Cohort). DRV, darunavir; DTG, dolutegravir; OSR, overall susceptibility rating; PDVF, protocol-defined virologic failure.

DTG (n=37)

OSR	At baseline			
At PDVF, n (%)	0 (n=21)	0.5 (n=6)	1 (n=9)	Missing (n=1)
0	15 (71)	2 (33)	3 (33)	1 (100)
0.5	0	2 (33)	0	0
1	0	1 (17)	5 (56)	0
Missing	6 (29)	1 (17)	1 (11)	0

OSR-new	At baseline			
At PDVF, n (%)	0 (n=28)	0.5 (n=4)	1 (n=5)	Missing (n=0)
0	27 (96)	2 (50)	3 (60)	0
0.5	0	1 (25)	0	0
1	0	1 (25)	2 (40)	0
Missing	1 (4)	0	0	0

DRV (n=34)

OSR	At baseline			
At PDVF, n (%)	0 (n=21)	0.5 (n=9)	1 (n=4)	Missing (n=0)
0	19 (90)	4 (44)	1 (25)	0
0.5	0	3 (33)	0	0
1	0	0	2 (50)	0
Missing	2 (10)	2 (22)	1 (25)	0

OSR-new	At baseline			
At PDVF, n (%)	0 (n=34)	0.5 (n=0)	1 (n=0)	Missing (n=0)
0	34 (100)	0	0	0
0.5	0	0	0	0
1	0	0	0	0
Missing	0	0	0	0

Change in score from baseline to PDVF

Decrease | No change | Increase

SUPPLEMENTAL SECTION S1

Predicted Antiretroviral (ARV) Activity of the Initial Optimized Background Therapy (OBT)

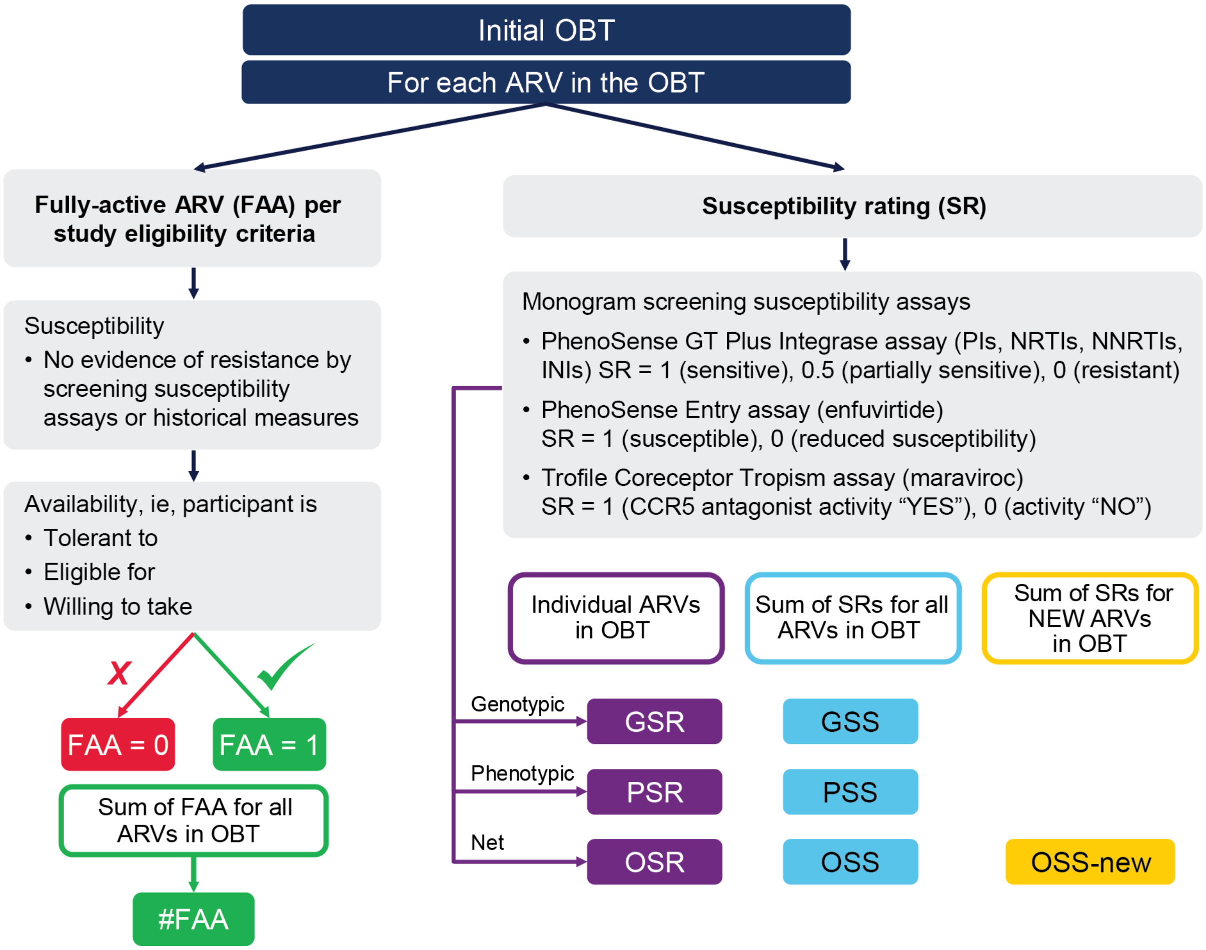

Number of Fully-Active ARVs

The number of fully-active ARVs (#FAA) in the initial OBT is the sum of fully-active ARVs in the initial OBT. #FAA for the initial OBT was recorded as unknown if at least 1 ARV in the initial OBT has unknown active status or if the initial OBT cannot be determined.

Baseline ARV resistance status was determined by per-protocol Monogram assays as follows:

- For drugs in the protease inhibitor (PI), nucleoside reverse transcriptase inhibitor (NRTI), non-nucleoside reverse transcriptase inhibitor (NNRTI), and integrase inhibitor (INI) classes using the PhenoSense GT Plus Integrase assay
  - Sensitive, if the net assessment is sensitive
  - Resistant, if the net assessment is partially sensitive or resistant
  - Not sequenced, if the net assessment is non-reportable or missing
- For enfuvirtide using the PhenoSense Entry assay
  - Sensitive, if the susceptibility assessment is sensitive
  - Resistant, if the susceptibility assessment is reduced susceptibility
  - Not sequenced, if the susceptibility assessment is non-reportable or missing
- For maraviroc using the Trofile Coreceptor Tropism assay
  - Sensitive, if CCR5 antagonist activity is yes
  - Resistant, if CCR5 antagonist activity is no
  - Not sequenced, if the CCR5 antagonist activity is non-reportable or missing

Historical ARV resistance status was determined from the Prior HIV Treatments CRF as follows:

- Sensitive, if medication resistance is sensitive or not applicable
- Resistant, if medication resistance is partially sensitive or resistant
- Not reported, if medication resistance is missing

ARV OBT availability status was determined from the Prior HIV Treatments CRF as follows:

- Available, if both of the following criteria are met:
  - Response to the lead question about OBT availability is yes
  - Responses to the 3 sub-questions (unwilling to take the ARV, intolerant of the ARV, ineligible for the ARV) are either no or missing
- Unavailable, if either of the following criteria are met:
  - Response to the lead question about OBT availability is no
  - At least 1 response to a sub-question is yes
- Not reported, otherwise

Experimental ARVs other than fostemsavir, such as ibalizumab, are counted as fully active, regardless of data reported on the Prior HIV Treatments CRF. Boosting agents such as ritonavir and cobicistat are excluded as fully active.

Monogram Genotypic and Phenotypic Susceptibility Scoring

The genotypic susceptibility score (GSS) was created by calculating a genetic susceptibility rating (GSR) for each ARV drug in the initial OBT and then summing up those ratings. The GSS was missing if at least 1 ARV drug in the initial OBT had a missing GSR.

For drugs in the PI, NRTI, NNRTI, and INI classes, the GSR for each drug was based on the baseline genotypic susceptibility (genotype) from the Monogram PhenoSense GT Plus Integrase assay. Non-protocol assays were excluded. The GSR for drugs in these ARV classes was calculated as follows:

- 1, if the genotype had evidence of drug sensitivity

- 0.5, if the genotype had evidence of partial drug sensitivity
- 0, if the genotype had evidence of drug resistance
- Missing, if the genotype was non-reportable or missing

For the fusion inhibitor (FI) enfuvirtide in the absence of a genetic test, the GSR was based on the Monogram PhenoSense Entry assay and was calculated as follows:

- 1, if the susceptibility assessment was sensitive
- 0, if the susceptibility assessment was reduced susceptibility
- Missing, if the susceptibility assessment was non-reportable or missing

For the CCR5 receptor antagonist (RA) maraviroc in the absence of a genetic test, the GSR was based on the Monogram Trofile Coreceptor Tropism assay and was calculated as follows:

- 1, if the CCR5 antagonist activity was yes
- 0, if the CCR5 antagonist activity was no
- Missing, if the CCR5 antagonist activity was non-reportable or missing

Experimental ARV drugs other than fostemsavir in the initial OBT were assigned GSR = 1. Boosting agents such as ritonavir and cobicistat were assigned GSR = 0. The GSR of tenofovir alafenamide fumarate (TAF) and tenofovir disoproxil fumarate (TDF) was based on the GSR of tenofovir.

The phenotypic susceptibility score (PSS) was created by calculating a phenotypic susceptibility rating (PSR) for each ARV drug in the initial OBT and then summing up those ratings. The PSS was missing if at least 1 ARV drug in the initial OBT had a missing PSR.

For drugs in the PI, NRTI, NNRTI, and INI ARV classes, the PSR for each drug was based on the baseline phenotypic susceptibility (phenotype) from the Monogram PhenoSense GT Plus Integrase assay. Non-protocol assays were excluded. The PSR for drugs in these ARV classes was calculated as follows:

- 1, if the phenotype had evidence of drug sensitivity
- 0.5, if the phenotype had evidence of partial drug sensitivity
- 0, if the phenotype had evidence of drug resistance
- Missing, if the phenotype was non-reportable or missing

For the FI enfuvirtide, the PSR was based on the Monogram PhenoSense Entry assay and was calculated as follows:

- 1, if the susceptibility assessment was sensitive
- 0, if the susceptibility assessment was reduced susceptibility
- Missing, if the susceptibility assessment was non-reportable or missing

For the CCR5 RA maraviroc, the PSR was based on the Monogram Trofile Coreceptor Tropism assay and was calculated as follows:

- 1, if the CCR5 antagonist activity was yes
- 0, if the CCR5 antagonist activity was no
- Missing, if the CCR5 antagonist activity was non-reportable or missing

Experimental ARV drugs other than fostemsavir in the initial OBT were assigned PSR = 1. Boosting agents such as ritonavir and cobicistat were assigned PSR = 0. The PSR of TAF and TDF was based on the PSR of tenofovir.

The overall susceptibility score (OSS) captures both genotypic and phenotypic assessments of susceptibility. The OSS was created by calculating an overall

susceptibility rating (OSR) for each ARV in the initial OBT and then summing up those ratings. The OSS was missing if at least 1 OSR was missing.

For drugs in the PI, NRTI, NNRTI, and INI ARV classes, the OSR for each drug was based on the baseline combination genotype/phenotype net assessment from the Monogram PhenoSense GT Plus Integrase assay. Non-protocol assays were excluded. The OSR for drugs in these ARV classes was calculated as follows:

- 1, if the net assessment was sensitive
- 0.5, if the net assessment was partially sensitive
- 0, if the net assessment was resistant
- Missing, if the net assessment was non-reportable or missing

For the FI enfuvirtide, the OSR was based on the Monogram PhenoSense Entry assay and was calculated as follows:

- 1, if the susceptibility assessment was sensitive
- 0, if the susceptibility assessment was reduced susceptibility
- Missing, if the susceptibility assessment was non-reportable or missing

For the CCR5 RA maraviroc, the OSR was based on the Monogram Trofile Coreceptor Tropism assay and was calculated as follows:

- 1, if the CCR5 antagonist activity was yes
- 0, if the CCR5 antagonist activity was no
- Missing, if the CCR5 antagonist activity was non-reportable or missing

Experimental ARV drugs other than fostemsavir in the initial OBT were assigned OSR = 1. Boosting agents such as ritonavir and cobicistat were assigned OSR = 0. The OSR of TAF and TDF was based on the OSR of tenofovir.

SUPPLEMENTAL SECTION S2

BRIGHTE Study Investigators

Argentina: P Cahn, L Cassetti, DO David, E Loiza, D Cecchini, S Lupo, M Martins, C Zala

Australia: A Carr, J McMahon

Belgium: S De Wit, E Florence

Brazil: CR Alves, J Andrade Neto, M Della Negra, R Diaz, B Grinsztejn, J Madruga, K Morejon, F Ribeiro, E Sprinz

Canada: M Murray, J Szabo, S Trottier, S Walmsley

Chile: J Ballesteros, F Zamora, C Beltran, C Chahin Anania, C Perez, M Wolff Reyes

Colombia: J Velez

France: PM Girard, C Katlama, J-M Molina, D Neau, G Pialoux, I Poizot-Martin, F Raffi, D Salmon-Ceron

Germany: K Arastéh, A Baumgarten, J Bogner, M Hower, W Kern, D Schürmann, C Stephan

Greece: S Metallidis, V Paparizos

Ireland: P Mallon

Italy: A Antinori, R Cauda, A Lazzarin, G Migliorino, C Mussini, G Orofino, G Rizzardini

Mexico: PF Belaunzaran, R Cabello, J Duque Rodríguez, M Santoscoy-Gómez, SC Treviño

Netherlands: I Hoepelman

Peru: F Mendo, Y Pinedo Ramirez

Poland: M Parczewski, B Knysz

Portugal: N Janeiro, F Maltez

Romania: L Preotescu, A Streinu-Cercel

South Africa: G Latiff, I Mitha

Spain: JM Llibre Codina, S Moreno Guillén, J Pineda

Taiwan: SM Hsieh

United Kingdom: A Pozniak

United States: J Aberg, J Bartczak, M Berhe, T Campbell, C Creticos, E DeJesus, V Drelichman, C Durand, J Eron, C Fichtenbaum, R Grossberg, S Gupta, F Haas, D Hagins, M Jain, M Kozal, P Kumar, J Lalezari, J Lennox, R Loftus, R Lubelchek, J McGowan, M McKellar, A Mills, J Morales-Ramirez, O Osiyemi, N Ramgopal, S Schrader, J Slim, P Tebas, M Thompson, W Towner, T Wilkin, A Wurcel
